# Toward diversity-responsive medical education: taking an intersectionality-based approach to a curriculum evaluation

**DOI:** 10.1007/s10459-015-9650-9

**Published:** 2015-11-24

**Authors:** M. E. Muntinga, V. Q. E. Krajenbrink, S. M. Peerdeman, G. Croiset, P. Verdonk

**Affiliations:** Department of Medical Humanities, EMGO Institute for Health and Care Research, VU University Medical Center, Amsterdam, The Netherlands; VU University Medical Center School of Medical Sciences, Amsterdam, The Netherlands; Department of Neurosurgery, VU University Medical Center, Amsterdam, The Netherlands; Department of Medical Humanities, EMGO Institute for Health and Care Research, School of Medical Sciences, VU University Medical Center, Amsterdam, The Netherlands

**Keywords:** Diversity, Diversity-responsiveness, Curriculum evaluation, Culture, Class, Gender, Intersectionality, Medical education

## Abstract

Recent years have seen a rise in the efforts to implement diversity topics into medical education, using either a ‘narrow’ or a ‘broad’ definition of culture. These developments urge that outcomes of such efforts are systematically evaluated by mapping the curriculum for diversity-responsive content. This study was aimed at using an intersectionality-based approach to define diversity-related learning objectives and to evaluate how biomedical and sociocultural aspects of diversity were integrated into a medical curriculum in the Netherlands. We took a three-phase mixed methods approach. In phase one and two, we defined essential learning objectives based on qualitative interviews with school stakeholders and diversity literature. In phase three, we screened the written curriculum for diversity content (culture, sex/gender and class) and related the results to learning objectives defined in phase two. We identified learning objectives in three areas of education (medical knowledge and skills, patient–physician communication, and reflexivity). Most diversity content pertained to biomedical knowledge and skills. Limited attention was paid to sociocultural issues as determinants of health and healthcare use. Intersections of culture, sex/gender and class remained mostly unaddressed. The curriculum’s diversity-responsiveness could be improved by an operationalization of diversity that goes beyond biomedical traits of assumed homogeneous social groups. Future efforts to take an intersectionality-based approach to curriculum evaluations should include categories of difference other than culture, sex/gender and class as separate, equally important patient identities or groups.

## Introduction

Systems of value related to health and wellbeing are largely influenced by a patient’s cultural background and social group membership (Napier et al. 2014). As awareness about the relationship between socio-cultural factors that underlie a patient’s health beliefs and practices and their health outcomes is growing, so is the need for physicians that are competent to provide adequate care to patients of different cultures and backgrounds (Rapp 2006; Napier et al. 2014). Research into this topic suggests that preparing physicians to meet the needs of a diverse population can enhance the quality of patient–doctor interactions, improve health outcomes of marginalized or minority demographics, and reduce health disparities between groups (Dogra et al. 2009; Awosogba et al. 2013; Maldonado et al. 2014). Medical education is considered an important terminal through which culturally competent and diversity-sensitive practices can be integrated into the health care system (Verdonk et al. 2009; Napier et al. 2014; Betancourt 2003). In recent years, governing institutions as well as researchers and educational specialists have recommended and even required medical schools to address diversity issues in their curricula (Dogra et al. 2009; Rapp 2006; Shaya and Gbarayor 2006; Li et al. 1998; Betancourt 2003). To realize optimal implementation of these issues, however, it has been suggested that medical education should be reformed at three interconnected levels: at a compositional level (i.e. the equal representation of minority groups and individuals of diverse backgrounds at all levels of health education and in the teaching staff), which is referred to as “fixing the numbers”, at an organisational level (i.e. an inclusive organizational and educational climate), referred to as “fixing the institution”, and at a curriculum level (i.e. embedding teaching content related to socio-cultural and biomedical aspects of diversity into preclinical and clinical programs), referred to as “fixing the knowledge” (Saha et al. 2008; Kennedy et al. 2008; Schiebinger 2008). Fixing the numbers, fixing the institution and fixing the knowledge are mutually reinforcing and equally important to achieve diversity-responsive medical education.

While experts agree that diversity deserves a prominent place on the medical educational agenda and medical schools are increasingly aiming to embed cultural diversity in their curriculum, opinions differ as to how cultural diversity should be defined and what it comprises. Literature that covers cultural diversity in medical education features both ‘narrow’ and ‘broad’ views on culture. The narrow definition of culture refers to factors such as ethnicity, nationality, language and migrant status, which implies the scope of the word culture to pertain to the values, beliefs, practices and customs of diverse ethnic groups (Kleinman and Benson 2006; Knipper et al. 2010). The broad definition is supported by authors who argue that culture involves more than variations in ethnic background and country of origin, and that other biological, social and cultural categories, such as gender and class, should be included in the definition (Young et al. 2012). For instance, Knipper et al. describe culture as “dynamic, changes over time, incorporates individual experiences and thus (culture) differs between people irrespective of their ethnicity” (Knipper et al. 2010). Napier et al. use a definition of culture that includes all social systems of belief as well as “presumptions of objectivity” (i.e. the positivist notion that biomedical knowledge is objective, impartial and universal) that influence perspectives on health and health care (Napier et al. 2014). Currently, medical schools use both the narrow and broad definition of culture to fix their numbers, institution, and knowledge.

Approaches to integrate cultural diversity at a curriculum level are informed by the nature of diversity knowledge focussed on by the medical school. Dogra (2003) distinguishes two: the ‘cultural expertise’ approach and the ‘cultural sensibility’ approach. The cultural expertise approach is grounded in biomedicine and thus fact-driven, whereas the cultural sensibility approach is located within a social constructivist perspective and aims to increase awareness of socio-cultural aspects of patient-provider encounters (Dogra 2003). Betancourt (2003) describes an additional third approach to diversity teaching, the cross-cultural approach, which focuses on the development of medical interviewing skills and tools to improve the patient–physician interaction (Betancourt 2003). The three approaches have different outcomes: the cultural expertise approach will primarily focus on factual medical knowledge, while the cultural sensibility and the cross-cultural approach aim to improve students’ critical reflexivity and communication competencies (Betancourt 2003; Dogra 2003). Together with their choice of operationalization of “culture”, a medical school’s approach to diversity teaching shapes faculty-level policy guidelines and diversity-related curriculum content.

### Mapping diversity

In the Netherlands, there has been an aim to outline central guidelines for diversity-responsive content. The Dutch Blueprint for final objectives of medical education (Laan et al. 2010) state that medical schools should adequately prepare their students to communicate with socio-culturally diverse patients, and that schools are required to educate students about the importance of taking into account gender, age, life style and cultural background when entering the process of prevention, diagnosis, treatment and prognosis (Laan et al. 2010). One of the Dutch medical schools that has been taking steps to implement these guidelines into their curriculum is the Amsterdam-based VU University Medical Center School of Medical Sciences (VUmc SMS) (Croiset 2013). In the VUmc SMS curriculum, the three approaches to teaching cultural diversity (i.e. the cultural expertise approach, the cultural sensitivity approach and the cross-cultural approach) coexist, which means that there is a focus on transferring knowledge about biomedical and socio-cultural aspects of diversity, as well as on the development of communication and critical reflexivity skills. To further optimize and implement diversity teaching, we identified a need for more insight in the extent to which cultural diversity issues were receiving attention in the VUmc SMS curriculum. One way of gaining such insight is by carrying out a curriculum evaluation, also referred to as ‘curriculum mapping’. Curriculum mapping allows faculties to provide detailed information about the structure and content of their education programs (Ellaway et al. 2014). As efforts to implement diversity topics increase, so does the need to map diversity-related content (Gustafson and Reitmanova 2010; Pena Dolhun et al. 2003; Betancourt 2003).

### Intersectionality

Evaluating how and to what extent diversity issues are implemented in the medical curriculum requires an analysis of how differences in health and health care issues between patient groups are addressed and explained. Three approaches are commonly used to analyse human difference: the unitary approach (that uses one factor or category of difference to understand a problem), the multiple approach (that adds multiple factors of difference to explain a problem rather than analysing relationships between these factors) and the intersectional approach (that considers the relationship between factors of difference and the processes that underlie them) (Hankivsky 2014). The intersectional approach is based on intersectionality, a critical strand of diversity theory that assumes that people occupy unique social locations based on multiple coexisting and mutually reinforcing social identities (such as gender, sexuality, social class, race and ethnicity), and that individual experiences attached to these locations reflect systems of oppression and privilege at a socio-structural level (Hankivsky 2012; Bowleg 2012). As intersectionality aims to analyse human experience beyond single categories of difference, it has been suggested that an intersectional approach is most qualified to critically investigate the complexity of multiple group similarities and differences, allowing evaluators to gain insight in the diversity-responsiveness of research, policy and practice (Hankivsky 2014). In addition, several authors have suggested that intersectionality can provide a more complex and dynamic framework for diversity teaching in medical education: by analysing how intersections of multiple socio-cultural and biosocial group memberships play a role in identity and health, students can increase their understanding of patients’ unique needs and experiences (Powell Sears 2012; Hankivsky et al. 2014; Hankivsky 2014; Tsouroufli et al. 2011). Considering these potential benefits of intersectionality for evaluation, teaching and learning, we took an intersectionality-based approach to evaluate the curriculum for diversity content and analyse our outcomes.

Similar to the unitary and multiple approaches, intersectionality uses categories of difference to analyse human identities and experiences. However, ideas differ as to whether any category-based methodology could ever fully grasp the complexity of human reality. McCall (2005) describes three main analytical stances toward the use of fixed categories in research of difference. *Intercategorical* researchers make strategic use of social categories (such as low-income women of color or homosexual men) to examine relationships of inequality and difference, and to explore how differences between categories vary depending on the context in which they exist. *Anticategorical* researchers theorize that reducing complex identities to any kind of fixed categories does not adequately represent the complexity of biological and socio-cultural processes that shape human identity. Anticategorical analysis is considered to lead to more inclusive social practices through the liberation of groups and individuals from normative and oppressive categories. Finally, *intracategorical* researchers neither reject nor fully embrace the use of categories: while critically questioning the benefits of boundaries, they use categorical analysis to study differences within categories with the aim to draw attention to the lived experiences of less visible social groups. The intracategorical approach is often used in case studies, where the aim is to perform an in-depth analysis of a single socio-cultural group or site (McCall 2005). As health outcomes are determined by sociocultural as well as biological and geographical factors, individuals do not only possess multiple social, but also multiple biosocial identities, e.g. identities determined by genetic predispositions, congenital status and exposure to determinants of disease. These biosocial identities of patients are continuous and dynamic (Epstein 2007) and intersect with sociocultural identities, resulting in a multidimensional continuum of unique biosociocultural locations. According to the anticategorical way of thinking, a categorical approach to analysing difference in health care would not suffice in representing the complexity and heterogeneity of patient experiences and bodies, regardless of the number of categories included in an analysis—in its worst case, it could induce essentialism and stereotypes, and prohibit person-centered care. However, abandoning categories altogether increases the chance that underexposed groups and the health and healthcare-related issues that may play a role in their lives remain obscure.

Since a major aim of our research was to critically assess diversity-related material for representations of different biosocial and sociocultural groups and their health risks and outcomes, the (intra and intercategorical) use of analytical categories allowed us to investigate to what extent the VUmc SMS curriculum was inclusive in addressing values, experiences and needs of a diverse range of patients. Although it has been suggested that using an intersectionality-based approach in medical education helps avoid essentialism (Powell Sears 2012), we nevertheless acknowledge that using fixed categories to investigate dimensions of difference could increase the risk of simplifying the complexity of lived experiences, and that such a method should always be used cautiously in order to prevent the homogenisation of patient experiences and bodies.

### Aim of the article

In this article, we report the results of an inventory of diversity-related curriculum content, performed at VUmc SMS and using an intersectionality approach. We asked the following research questions: (1) What are stakeholder’s opinions and ideas about embedding diversity in the VUmc SMS curriculum? (2) What are criteria for a diversity-responsive curriculum? (3) How is diversity currently addressed in VUmc SMS curricular content? To answer these questions, we explored VUmc SMS stakeholder’s ideas about diversity-responsive medical education, outlined learning objectives for diversity-responsive medical education, and mapped the written curriculum to gain insight in how cultural diversity issues, in particular issues regarding culture (ethnicity, nationality and religious background), sex/gender (including non-binary notions of gender), and class, are currently integrated within the VUmc SMS medical curriculum. Finally, we identified opportunities for further integration of diversity content. In this paper, we provide an example of how intersectionality can be used as an analytical foundation to evaluate the diversity-responsiveness of a medical curriculum. In addition, we propose intersectionality theory as an essential framework for designing diversity-related curriculum content. We hope to contribute to existing knowledge about opportunities and challenges that go along with integrating and managing diversity issues in medical schools.

## Methods

### VUmc SMS curriculum

VUmc SMS reaches a large population of minority students and patients. In the academic year 2009–2010, the total number of medical student was 2462, of which 20.1 % was of non-native Dutch background (Leyerzapf et al. 2014). More recent statistics are unavailable, as registering patients’ and students’ ethnic background meets cultural resistance and is complicated by Dutch law. The curriculum of VUmc SMS consists of a 3-year preclinical and a 3-year clinical program (i.e. bachelor and master). Like many other medical schools across Europe that have implemented a bachelor–master structure after the Bologna Declaration, the curriculum is generally vertically integrated (Patricio and Harden 2010). The preclinical program consists of basic medical sciences, practical and clinical skills, and professional development, whereas the clinical program centers on further improving students’ understanding of the basic sciences, and further development of their clinical and communication skills (Patricio and Harden 2010).Where possible, medical knowledge is interlaced with professional development (PD) by means of an overarching 6-year longitudinal educational domain. The domain consists of several themes, such as ‘Patient Safety’, ‘Communication’, ‘Ethics and Law’, and ‘Interculturalization and Diversity’, each theme addressing several CanMeds competencies (Mak-van der Vossen et al. 2013). The theme ‘Interculturalization^1^ and Diversity’, grounded in the Dutch Blueprint for final objectives of medical education (Laan et al. 2010) relates to the importance of diversity-responsive education to the various physician roles. For instance, the role of medical expert asks a physician to be able to apply her diagnostic, therapeutic, prognostic and preventive skills in an effective and morally responsible way, which in turn requires taking into account gender, age, life stage, and cultural background (Laan et al. 2010).

### Study design and sample

We combined aspects of a pragmatic design with aspects of a case study design: by using the object of study (VUmc SMS) as an analytical frame, we explicate ‘cultural diversity’ through the perspective of a particular medical school, producing local knowledge in the process (Yin 2013). The study consisted of three phases, carried out in consecutive steps: an exploratory phase, consisting of qualitative interviews with stakeholders, an interpretive phase, in which we identified learning objectives for a diversity-responsive medical curriculum, and a curriculum evaluation phase, in which we mapped the written curriculum for diversity-related content.

### Data collection and procedures

#### Exploratory phase

The aim of the exploratory phase was to gain more insight in key VUmc SMS stakeholders’ ideas regarding diversity-responsiveness in medical education by carrying out semi-structured interviews. Stakeholders (N = 8) were recruited through the Director of Education and the Head of the Medical Humanities Department by means of purposive sampling, and were considered eligible for inclusion if their position within the VUmc SMS organisation allowed them to directly influence diversity-specific educational policy and practice. All stakeholders accepted the invitation to be interviewed. The sample consisted of teachers and researchers, education coordinators and educational policymakers. The interviews were carried out in 2011 by PV and lasted between 30 and 60 min each. The following topics were addressed: perceptions of cultural diversity, aims with regards to diversity-responsive medical education, strategies for further integration and mainstreaming, and perceived barriers for further integration and mainstreaming. During the interviews PV made notes and wrote an interview report. All respondents received a summary of the report and were asked for feedback (member check), and reports were adjusted accordingly.

The interviews were analysed using framework analysis. Framework analysis is a deductive approach in which some of the research questions are predetermined, and has an overt orientation towards generating policy and practical strategies (Green and Thorogood 2014). In order to achieve intersubjective agreement, two authors (PV and MM) analysed the data separately. First, PV and MM familiarized with the data. Second, they formulated labels (both top-down and bottom-up) which they systematically applied to the data (indexing). Third, they rearranged data according to themes that emerged from the data.

#### Formative phase

In step 2, we outlined criteria for a diversity-responsive VUmc SMS curriculum. We defined criteria for a diversity-responsive curriculum as the learning objectives that should be addressed in educational material in order for diversity to be fully integrated in the curriculum. The learning objectives were based on the outcomes of themes that emerged from the interviews in step 1 and the existing literature. A policy document containing (1) the preliminary learning objectives for a diversity-responsiveness curriculum and (2) information about the way in which the objectives were established was sent to VUmc SMS stakeholders, who were asked to give feedback. After taking this feedback into account, the final learning objectives for diversity-responsiveness were established.

#### Curriculum mapping phase

The curriculum mapping was carried out as follows: From May to July 2013, we collected preclinical and clinical education material (i.e. module manuals, practical and theoretical assignments and lecture slides) from a digital learning environment. We then made a matrix of all materials that included at least one of the learning objectives for diversity -responsive content, and assigned the following labels to the material based on one or more of the following categories of difference: culture (comprised of the categories ethnicity, nationality and religious background, in this paper referred to as culture), sex/gender (comprised of the categories sex, gender, and sexual orientation, in this paper referred to as sex/gender), and class (comprised of categories of socioeconomic status, including income and education level, in this paper referred to as class). Separately from one another, two researchers (PV and VK) assessed whether culture, sex/gender and/or class was implicitly or explicitly mentioned in relation to one of the three earlier defined learning objectives, whether this was unclear, or whether it was not mentioned at all. Material was labelled ‘explicit’ when: (1) issues related to culture, sex and gender or class were a primary focus, (2) the material distinctly addressed the relationship between health, health care and diversity, and (3) learning objectives regarding diversity were clearly formulated. Material was labelled ‘implicit’ when (1) diversity was addressed but not a primary focus, and (2) learning objectives regarding diversity were not formulated or ambiguously formulated. Consensus was reached when material was labelled ‘doubtful’ (i.e. when it was unsure whether to choose implicit or explicit) or when researchers disagreed about a label. Material was excluded from analysis when the objective was unrelated to learning about diversity. After all the material was labelled, we investigated whether material had been assigned multiple labels (for instance both culture and sex/gender). Within this material, we analysed each category for the presence of other intersecting categories. For instance, we investigated whether a module (i.e. 2-h small-group practicals or tutorials) about cultural aspects of health or healthcare also specifically addressed issues related to the intersection of these cultural aspects with sex and gender or class.

## Results

### Exploratory phase: stakeholders’ ideas about diversity-responsiveness

The following three themes emerged from the interviews with stakeholders carried out in the exploratory phase: relevance of a diversity-responsiveness curriculum, essential diversity learning objectives, and implementation of diversity content in the curriculum.

#### Relevance of a diversity-responsive curriculum

All stakeholders acknowledged the importance of a diversity-responsive curriculum. They mentioned both pragmatic and ethical arguments. Stakeholders argue that diversity sensitivity is an essential aspect of medical professionalism, and that increasing students’ knowledge about diversity may help prevent negative health outcomes and increase quality of care. Stakeholders also considered diversity important because of its priority status in VUmc SMS’s education policy. From a social justice point of view, stakeholders felt that diversity responsiveness is important to target unequal access to professional opportunities for minority medical students, and that every patient has a right to receive tailored care from competent doctors. However, they mentioned that implementing diversity-responsive practices in the health care system should be efficient, i.e. resulting in less costs, better patient outcomes, or both.

#### Essential diversity learning objectives

Stakeholders believed that medical curricula should provide opportunities to explore diversity-related themes both in-depth and broadly, and that teaching content should reflect contemporary issues in health care and society. No consensus existed as to whether cultural diversity should be operationalized by a broad or narrow use of culture. However, all considered it important that students have knowledge of sociocultural and demographic characteristics of social groups and minority groups in the Netherlands, including value systems, health beliefs and health practices, and the skills to optimize communication with patients from varying socio-cultural backgrounds. They furthermore believed students should learn about social justice issues and socio-political concepts, such as oppression, stigma, exclusion and discrimination. Reflexivity training was considered essential to increase students’ awareness of their own cultural background and its role in patient–doctor interactions, and to provide them with the skills to critically reflect on their own assumptions about culture and cultural ‘others’. Stakeholders warned that an essentialist perspective on biomedical and cultural traits of social groups in written or oral content could lead to the ‘othering’ or stereotyping of groups of (minority) patients.

#### Barriers to implementation

Barriers to implementation of diversity content in the curriculum were perceived at a student, teacher and institutional level. For instance, stakeholders experienced that both students and teachers generally consider diversity-related topics uninteresting and irrelevant for medical practice. They felt that support at a student and teacher level can be improved by partnering with enthusiastic and influential early student adopters, by designing curricular material together with diversity competent physicians, and by organizing ‘teach the teacher’ courses. Furthermore, stakeholders mentioned that classes are taught by many different teachers and on many different locations, which impedes implementation. Implementing diversity content was considered more challenging in the clinical program than in the preclinical program. A centralized approach to designing and editing course material, stakeholders suggested, is more likely to secure a high degree of implementation. Overall, stakeholders shared the opinion that diversity mainstreaming should not only take place in the preclinical and clinical programs, but across the organisation. They highlighted the importance of creating equal access to opportunities for students and the need for a focus on cultural competent patient care. All acknowledge the need for a sound evaluation of mainstreaming efforts.

### Formative phase: essential learning objectives of a diversity-responsive curriculum

The process toward becoming a diversity-responsive physician involves the development of an orientation that recognizes dignity and autonomy of patients, and of a focus on providing high quality of care to a pluriform society across aspects such as culture, gender and class (Kumagai and Lypson 2009; Verdonk and Abma 2013). Based on this notion, we formulated three primary, overarching learning objectives, which we considered essential to implement in order to achieve a diversity-responsive curriculum. Objectives were related to the following areas of medical education: medical knowledge and skills, patient–physician communication, and reflexivity. We aimed to formulate the objectives in such a way so as to discourage essentialist or fixed perceptions of social groups or categories and their value systems, health practices and health outcomes.

The learning objective ‘medical knowledge and skills’ comprises the knowledge base essential for physicians to adequately approach diversity. We distinguished the following categories: specific conditions, chronic diseases, management and lifestyle, mental health and development, sexual health, reproductive health and society, and background determinants of health & health care use. The objective explicitly addresses the three “master identities” culture (ethnicity, nationality—including migrant status and language—and religious background), sex and gender, and class. First, students must be able to recognize and explain relevant differences between the five largest cultural groups in the Netherlands (i.e. Dutch, Dutch-Turkish, Dutch-Moroccan, Dutch-Surinamese and Dutch-Antilles) with regards to epidemiology, aetiology, presentation, diagnostics and treatment of illness and disease, and have insight in determinants of health disparities and inequalities between these groups. Second, students must understand and explain the relationship between sex or gender and health behaviour and outcomes. By using the term sex as well as gender, we refer to differences between women and men that are both biomedical (e.g. symptom presentation in cardiovascular disease) and sociocultural (e.g. risk of exposure to violence). Age, such as life-stages, and sexual orientation-related topics were listed under this objective. Third, students must have insight in present-day medical and social themes related to socioeconomic status and health, including class-based inequalities (e.g. differences in life expectancy and chronic diseases), occupational health, and (health) literacy.

The learning objective ‘patient–physician communication’ pertains to the skills necessary to adequately communicate with patients from diverse sociocultural backgrounds. Since adequate communication skills are necessary to provide high quality of care, students must be aware of the influence of both a patient’s and physician’s background on physician–patient communication. For instance, they must recognize the impact of language barriers and limited health literacy on outcomes of interactions, and develop competencies with regards to working with both professional and informal interpreters. In addition, students must understand that patients from various cultural backgrounds value different manifestations of the patient–physician relationship, and show the confidence to explore and respect patients’ relational expectations and preferences.

The final learning objective, ‘reflexivity’, involves critical thinking or self-reflection. Through self-reflexivity training, students acquire the skills to take on a critical attitude towards oneself. Such skills help them recognize their own prejudices towards patients who do not share their own sociocultural background, which is essential in order to preserve patients’ dignity and autonomy and deliver high-quality, personalized care in a pluriform society. The final definitions of the learning objectives are presented in Table 1.

**Table 1:** Three learning objectives for a diversity-responsive curriculum

Area of medical education	Learning objectives
Medical knowledge and skills	(1) The student is able to recognize and explain differences between cultural groups with regards to specific conditions, chronic disease, self-management and lifestyle, psychosocial complaints and psychiatry, sexual health and sexuality, and background determinants of health and health care use (2) The student is able to recognize and explain sex and gender differences with regards to life stages such as menopause and adolescence, specific conditions, chronic disease, self-management and lifestyle, psychosocial complaints and psychiatry, sexual health and sexuality, and background determinants of health and health care use (3) The student is able to recognize and explain socioeconomic/class differences with regards to children’s growth and development, chronic disease, self-management and lifestyle, psychosocial complaints and psychiatry, and background determinants of health and health care use
Patient–physician communication	(1) The student is aware of cultural differences in communication and physician–patient interaction and is able to adequately communicate with non-majority patients (2) The student is aware of gender differences in communication styles and in physician–patient interaction, and has the competency to adequately communicate with male and female patients (3) The student is aware of class issues in communication in general and health literacy in particular, and has the competence to adequately communicate with patients of different health literacy levels
Reflexivity	The student has an open attitude toward patients with a cultural background different from their own, is able to deliver culturally competent and gender specific health care, and is critically conscious with regards to diversity aspects in health and illness

### Curriculum evaluation phase

Table 2 presents the outcomes of the curriculum mapping. Most prevalent were modules related to clinical aspects of a patient’s ethnic, national or religious background (i.e. material with the label ‘culture’). Class issues were least often addressed. Diversity content included both biomedical and sociocultural aspects of health, which were most often addressed by means of single categories of difference (i.e. by either culture, sex/gender, or class). When material did address multiple categories, intersections between categories of difference remained often unexplored. Most diversity topics were addressed in the preclinical program. Integration of the three categories of difference and their intersections in the clinical program were challenging to investigate: since most teaching takes place during rotations, written material is limited.

**Table 2:** Results of curriculum screening for diversity content in the domain of medical knowledge and skills, stratified for culture, sex/gender and class

Medical knowledge and skills	Culture	Sex/gender	Class
Specific conditions	Vitamin D-deficiency, sickle cell disease, thalassemia, tropical communicable diseases, tuberculosis, malaria, familial Mediterranean fever^a^	Adolescence (endocrinology, pathology, neurology, genetics, sociocultural factors), pregnancy (pathology), menopause, menstrual cycle, dysmenorrhoea, amenorrhoea, UTI’s, UTI’s and UI during pregnancy, pharmacotherapy during pregnancy and lactation, physical examination^a^	Childrens’ growth and development^a^
Chronic diseases, self-management and lifestyle	Diabetes, health promotion and lifestyle (smoking, exercise, nutrition)^a^ Addiction (alcohol)^b^	Breast cancer, testicular cancer, prostate cancer, cervical cancer (pathology)^a^ Chronic pain^b^	Life expectancy (DALY’s and QALY’s), lifestyle (smoking, exercise, nutrition)^a^ Physical incapacity and chronic diseases such as lung disease, cardiovascular disease, diabetes, back pain, rheumatoid arthritis^b^
Mental health and development	Presentation of illness, interpretation of symptoms, disease experience (including pain), psychiatric conditions (depression, schizophrenia), coping with a physical disability (deafness)^a^ Psychosocial complaints, adoption and child development^b^	Eating disorders and obesity, depression and anxiety disorders, Posttraumatic stress disorders^a^ Addictions: alcohol, benzodiazepines^b^	Exposure to unfavourable working conditions^b^
Sexual health, reproductive health and society	Sexuality and culture of sexuality (marital practices, virginity, circumcision, genital mutilation), sexual health (HIV/STI’s)^a^ Reproductive health (infertility and subfertility, contraception, unwanted pregnancy and termination of pregnancy, single motherhood), prevention (cervical cancer screening/HPV-vaccination), sexual violence, consanguinity and genetic diseases in offspring, child abuse^a^	Male and female sexuality, sexual functioning and sexual dysfunction, reproduction and fertility, reproductive health (contraception, sexually transmitted infections, HPV, abortion), sexual identity (homosexuality), gender identity and gender dysphoria, paraphilias, child abuse^a^ Sexual violence^b^	
Background determinants of health and health care use	Access to health care, patient’s context, health of undocumented migrants, ethical issues and dilemmas (autonomy), alternative medicine^a^ Religious customs and health (e.g. Ramadan and fasting), end-of-life care, palliative care, family and informal care^b^	Gender and culture/ethnic background, undocumented migrants^a^ Occupational health and disease, informal care, end-of-life care, palliative care^b^	Health literacy^a^ Explanations for socioeconomic health inequalities, selection and causation, class and work, public health, socioeconomic health inequalities^b^
*Communication*	Language barriers^a^ Interpreters, telephone interpreter^b^	Sexual harassment in patient–physician relationship, sexual harassment at work, discussing sexuality and sexual functioning with patients Communication during andrological and gynaecological examination^a^	^c^
*Reflexivity*	Learning goal “The student has an open attitude towards patients with a non-Dutch cultural background”^a^ Learning goal “The student is able to deliver culturally competent health care/quality of care”^a^ Learning goal “The student is critically conscious with regards to diversity aspects of health and illness”^a^	Learning goal “The student offers gender specific health care/quality of care”^a^ Learning goal “The student is critically consciousness with regards to diversity aspects of health and illness”^a^	^c^

^a^Category or learning objective explicitly included

^b^Category or learning objective implicitly included

^c^Category not included

#### Good practices

Our findings show that the VUmc SMS curriculum featured several good diversity teaching practices. First, students learned about aetiology, pathology, physiology and epidemiology of specific diseases related to ethnicity and geographic location, such as communicable diseases and tropical infections (e.g. tuberculosis, amoeba infection), genetic dispositions (e.g. familial Mediterranean fever), and determinants of disease. A year-one preclinical module addressed complementary and alternative medicine (CAM). Reading material included chapters of a textbook addressing the role of cultural and social factors in health and disease (i.e. Helman C.G., Culture, health and illness: An introduction for health professionals. Butterworth-Heinemann). In communication modules that took place throughout the preclinical program, students were trained to communicate with patients of diverse cultural backgrounds. Self-reflexivity related to culture was addressed in a module about the culture of Western medicine. In addition, we identified good practices related to sex/gender and class. Sex differences in heart disease were addressed in preclinical year 3, and gender was a focus in preclinical modules that addressed sexual harassment and sexual abuse, including child abuse. Class-related health inequalities were addressed in preclinical year 1 and 2 through modules on SES and education level-based differences in life expectancy, health literacy, and working conditions. Diversity aspects were also incorporated in preclinical and clinical internship programs. Good practices were a preclinical reflection assignment about cultural diversity, and intervision sessions for year-two clinical students where, among other topics, experiences with culture-related aspects of diversity were addressed. Finally, we identified a few modules that took an intersectional approach to analysing and explaining health outcomes. In preclinical year two, for instance, a module on malnutrition addressed the intersections of ethnicity and poverty in relation to vitamin D-deficiency in children who receive long-term breastfeeding. Another preclinical module about health care for undocumented migrants addressed the intersections of migrant status, being undocumented and gender, and their relation to health outcomes and access to health care.

#### Opportunities for improvement

Several learning objectives were absent or only marginally addressed. For instance, we found few material that explicitly dealt with diversity in relation to lifestyle issues, self-management and determinants of chronic disease. Communication and reflexivity were only marginally addressed from a diversity perspective. In addition, several diseases (Behçet’s disease and brucellosis) and communication topics (working with interpreters, consultations with religious leaders) remained completely unaddressed. Furthermore, sex and gender differences related to diseases of non-sexed organ systems or chronic conditions (including auto-immune diseases) received little attention, and pharmacological sex differences were discussed only in reference to teratogenicity. Partner violence was not addressed at all. Moreover, sex was presented as a stable category, and gender was used as a fixed binary. Communication-related content did not address gender differences relevant for the patient–physician communication, as was the case with reflexivity-related content. Finally, there was a limited focus on class-related determinants of health outcomes; class-related factors such as health literacy were not mentioned in relation to physician–patient communication, and class was not included in reflexivity assignments. We found no written material on diversity among ageing populations and the ageing process. Only a few modules explicitly addressed intersections of culture, sex/gender and class—most often, diversity issues were analysed without taking interactions between biosocial and/or sociocultural group memberships into account at all, or analyses centered on the experience of a single social group. For instance, there was no mention of the role of gender or class in material on sexual violence, and the gendered topic ‘abortion’ was presented from a white, native-Dutch, middle-class perspective.

## Discussion

In this paper, we reported the outcomes of a multiple method study that took place at an Amsterdam, the Netherlands-based medical school, VUmc SMS. We explored local ideas about diversity-responsive medical education, outlined diversity-responsive learning objectives for preclinical and clinical medical education, and mapped the VUmc SMS curriculum for diversity-responsive material using an intersectionality-based approach.

### Toward diversity-responsive medical education

Outcomes of the curriculum mapping expose that, despite several good practices, diversity-responsiveness of the curriculum could be improved. For instance, most content referred to a narrow definition of culture (i.e. ethnicity, nationality, religious background and migration history) in the area of medical knowledge and skills. Class remained the least pronounced aspect of diversity despite its strong association with life style and life expectancy (Mackenbach et al. 2008). Diversity issues in patient–physician communication could receive more focus in the curriculum; authors have suggested that in order to adequately explore and address differences in patients’ beliefs bout disease, health and treatment, medical students require the skills to be sensitive in their communication, adjust their style of communication, and actively target language barriers, for instance by using an interpreter (Betancourt 2003; Kutob et al. 2013; Knipper et al. 2010; Suurmond et al. 2011). While critical reflexivity with regards to students’ own cultural background and identity was largely absent from written curriculum material, experts have claimed that diversity-responsive medical education should involve the fostering of a critical awareness—a critical consciousness—of the self, others, and the world, and a commitment to addressing issues of societal relevance in health care (Kumagai and Lypson 2009; Verdonk 2015). Kumagai and Lypson (2009) describe critical consciousness and posit that it is different from, albeit complementary to, critical thinking. Acquiring critical thinking and critical consciousness skills can help students learn to reflect on their own norms, values and position; when possessed by physicians, such skills may contribute to more health equity (Verdonk and Abma 2013). Critical consciousness, however, cannot be achieved without an increased awareness of the context in which students’ norms and values exist, and in the systematic issues that determine and sustain differences in health outcomes. To avoid non-performativity of reflexivity teaching, diversity-responsive curricula, therefore, should offer students insight in how the production and reproduction of power at a socio-structural level shapes relationships between groups and individuals, and how dominant paradigms of health and illness and the hegemony of medical knowledge shape patients’ lived realities and bodies (Verdonk 2015).

Largely lacking from the curriculum was content that explored determinants of health and patient experiences based on the unique biosocial and sociocultural locations of patients. Instead, the role of biosocial and sociocultural factors in health outcomes was addressed by means of single categories of difference. This suggests a unitary or multiple approach to diversity teaching (Hankivsky 2014). Although such approaches may help improve students’ understanding of shared group characteristics and experiences, it could easily lead to essentialism when not addressed critically (Powell Sears 2012; Hankivsky et al. 2009). Taking an intersectional approach to medical education may avoid essentialism and deepen students’ understanding of human difference. Analysing health-related issues from multiple and more complex perspectives can enhance students’ understanding of patients’ value systems, lived experiences and health and care needs. By means of its focus on systems of oppression and privilege and their influence on human experience (both at a societal and a health system level), intersectionality theory may contribute to an increased visibility of marginalized and/or minority groups in health education and health care. Along these lines, adopting a methodology that uses either an intercategorical approach to difference (analyses between groups), an intracategorical approach to difference (analyses within groups) or both can document the degree to which marginalized or minority patient groups receive attention in educational content (McCall 2005). However, as mentioned in the Methods section of this paper, we believe that all categorical approaches to human health identity will inevitably struggle to grasp the complexity of lived patient experiences and the social and biosocial processes that produce and produce them, let alone succeed to provide the information to translate this complexity to medical know-how. The awareness that using categories are needed but do not provide an accurate model of identity and difference [described by Epstein as a ‘split consciousness’ (Epstein 2007)] may therefore be a diversity competency in and of itself, one that students may or may not develop through reflexivity training and everyday medical practice.

### Moving beyond fixing the knowledge

While taking an intersectionality approach may contribute to more diversity-responsive curriculum content, organizational reform should move beyond ‘fixing the knowledge’ by making diversity responsiveness an integral aspect of the organisation through an additional focus on ‘fixing the institutions’ and ‘fixing the numbers’. Fixing the institutions requires that medical schools should aim to establish compositional diversity (i.e. a composition of student, teaching staff and hospital staff demographics that reflects the general population composition) which requires an active monitoring of numerical representation of, for instance, women and ethnic minorities at different hierarchical levels, and a special focus on creating equal opportunities for underrepresented groups (Dogra et al. 2009). Fixing the numbers requires fixing the institution, for instance by enhancing a school’s inclusivity through policies targeting exclusion and oppression, such as policies addressing harassment, sexual violence, discrimination and racism, or by the implementation of measures to eliminate biased procedures for student recruitment, selection and performance evaluation (Rademakers et al. 2008; Powell Sears 2012; Leyerzapf et al. 2014; Zimmerman and Hill 2000; Leyerzapf et al. 2014). The aim for an inclusive learning climate also demands an effort towards interactional diversity, which refers to the stimulation of positive interactions between students with diverse backgrounds (Saha et al. 2008). As fixing the numbers, fixing the institutions, and fixing the knowledge are interdependent and complementary, they cannot be seen as isolated domains of institutional reform. For example, sociocultural issues are more likely to be integrated into the curriculum by a medical school with a larger cultural diversity among their student body (Van Wieringen et al. 2003; Verdonk et al. 2009).

However, the transformative potential of fixing the knowledge, institution and numbers is largely mediated by the acceptance of cultural diversity issues as legitimate medical knowledge. Although the inclusion of non-medical physician roles in official competency frameworks suggests that knowledge about sociocultural aspects of health and healthcare is considered as foundational for medical practice as traditional biomedical knowledge (Kuper and D’Eon 2011), the historical lack of teachers’ and students’ exposure to social science paradigms within medical education has influenced expectations of what type of content should be featured in a curriculum that adequately prepares future doctors for practice. Hierarchical notions of knowledge can hinder effective implementation of diversity issues, as outcomes of interviews in Phase 1 support. In order to overcome this barrier, increasing teachers’ and students’ understanding of the relationship between diversity knowledge and physician competencies is essential (Kuper and D’Eon 2011).

### Study strengths and limitations

This study has several strengths and limitations. First, by analyzing content at the level of knowledge, communication and reflexivity, we took into account the three approaches to diversity teaching (i.e. the cultural expertise, the cultural sensibility and the cross-cultural approach), acknowledging both the knowledge-base of diversity (such as knowledge about particular diseases in relevant social groups), the pivotal role of communication in successful patient-provider interactions, and the importance of awareness of health professionals’ own perspectives and how they affect health care delivery (Betancourt 2003; Dogra 2003). However, as Dogra (2003) mentions, the different outcomes of these approaches have consequences for assessment. We experienced that mapping the written curriculum did not suffice to evaluate the degree of implementation of cultural sensibility or critical thinking (Fook and Askeland 2007; Dogra 2003). Other study designs and methods are needed to provide such insight.

Second, while we did take into account factors of difference other than culture, sex/gender and class when mapping the curriculum, we did not include them as separate categories of difference. Instead, such factors were categorized under one of the aforementioned three categories. For instance, sexual orientation and age were categorized under sex/gender, and ableness was categorized under culture. We suggest that future efforts to take an intersectionality-based approach to curriculum mapping aim to include other categories of difference as separate, equally important patient identities or groups. An evaluation of the representation of these identities is particularly necessary since they are often underrepresented or absent in medical curricula. For instance, research has shown that time spent on LGBT-issues was small across curricula in the US, Canada and South-Africa, and that the quality, quality and content of educational material varied (Muller 2013; Obedin-Maliver et al. 2011). To increase quality of care for patients at intersections of less visible or marginalized categories, future research in this area should focus on exploring whether and how their issues are addressed in medical curricula.

Third, since students’ acceptance of knowledge about diversity as useful for practice negotiates the degree to which diversity issues are considered legitimate by faculty staff, teachers and peers, they can be considered active stakeholders in the process toward a diversity-responsive curriculum. Therefore, parallel to Phase 1 of this study, another study was carried out that explored medical students’ experiences with diversity issues within the VUmc SMS curriculum, results of which were reported in a Dutch language paper (Tjitra et al. 2011). The authors of the paper reported that students with a minority background experienced a lack of respect in the way they were treated by peers and teachers (for instance during modules on physical examination), and that these students felt that patient cases used to teach about specific health issues of minority groups were stigmatising and stereotypical (Tjitra et al. 2011). As this provided us with insight in the student perspective, we chose not to interview students as part of this study. However, in the context of the subject matter addressed in this paper, we consider not including the student perspective a limitation.

## Conclusions and recommendations

In order for diversity issues to be put on the medical education agenda, endorsement at schools’ administrative and political level is essential (Verdonk et al. 2009). At Vumc SMS, diversity-responsiveness is an explicit focus of the school’s and the university’s education policy (Croiset 2013; Vrije Universiteit Amsterdam 2014; Verdonk 2013). Outcomes of qualitative interviews with stakeholders show that the topic receives widespread support. While this suggest that important prerequisites for diversity-responsiveness are met, results of the curriculum screening indicate that the diversity-responsiveness of teaching material could be improved. Even in a supportive climate where diversity teaching and learning is favoured, curriculum material does not necessarily reflect top-down policy statements.

So how can we move forward? We believe that fixing numbers, institutions and knowledge should happen simultaneously. Achieving a high degree of diversity-responsiveness in curricula and organisations requires the active participation of multiple actors (teachers, students) and a shared drive for change and innovation. Attention to diversity in policy is therefore a start but not an end game, and implementation across schools may require practice-based efforts and workforce-directed programs. Teacher trainings, for instance, can help educators teach about patient diversity, improve education material, and manage diverse classrooms in a safe and inclusive way (De Jong 2014; Knipper et al. 2010). Performance achievement and application and selection procedures should be reassessed to secure equal access to professional opportunities for students (Young et al. 2012).

With this paper, we hope to contribute to the existing knowledge about using curriculum mapping to investigate the diversity-responsiveness of written medical curriculum content. With increasing demands put on physicians to provide tailored care to a diversifying patient population, and with limitations put on the quantity of diversity teaching hours due to saturated programs, high-quality education material, a skilled workforce and diversity-oriented school policies are essential ingredients to achieve diversity-responsive medical education—and to eventually achieve diversity-responsive health care.

## Footnotes

1 Interculturalisation is a transformative process within an organisation that aims to adjust resources and services to the needs of a pluriform society through structural reform of social practices.

## References

[CR1] Awosogba T, Betancourt JR, Conyers FG, Estape ES, Francois F, Gard SJ (2013). Prioritizing health disparities in medical education to improve care. Annals of the New York Academy of Sciences.

[CR2] Betancourt JR (2003). Cross-cultural medical education: Conceptual approaches and frameworks for evaluation. Academic Medicine.

[CR3] Bowleg L (2012). The problem with the phrase women and minorities: Intersectionality—an important theoretical framework for public health. American Journal of Public Health.

[CR4] Croiset G (2013). Onderwijs: een Kunst!.

[CR5] Dogra N (2003). Cultural competence or cultural sensibility? A comparison of two ideal type models to teach cultural diversity to medical students. International Journal of Medicine.

[CR6] Dogra N, Reitmanova S, Carter-Pokras O (2009). Twelve tips for teaching diversity and embedding it in the medical curriculum. Medical Teacher.

[CR7] Ellaway RH, Albright S, Smothers V, Cameron T, Willett T (2014). Curriculum inventory: Modeling, sharing and comparing medical education programs. Medical Teacher.

[CR8] Epstein S (2007). Inclusion: The politics of difference in medical research.

[CR9] Fook J, Askeland GA (2007). Challenges of critical reflection: ‘Nothing ventured, nothing gained’. Social Work Education.

[CR10] Green J, Thorogood N (2014). Qualitative methods for health research.

[CR11] Gustafson DL, Reitmanova S (2010). How are we ‘doing’ cultural diversity? A look across English Canadian undergraduate medical school programmes. Medical Teacher.

[CR12] Hankivsky O (2012). Women’s health, men’s health, and gender and health: Implications of intersectionality. Social Science and Medicine.

[CR13] Hankivsky O (2014). Intersectionality 101. cal.

[CR14] Hankivsky O, Cormier RE, DeMerich D (2009). Intersectionality: Moving women’s health research and policy forward.

[CR15] Hankivsky O, Grace D, Hunting G, Giesbrecht M, Fridkin A, Rudrum S (2014). An intersectionality-based policy analysis framework: Critical reflections on a methodology for advancing equity. International Journal of Equity in Health.

[CR16] Jong D (2014). Diversiteit in het hoger onderwijs.

[CR17] Kennedy HP, Fisher L, Fontaine D, Martin-Holland J (2008). Evaluating diversity in nursing education: A mixed-method study. Journal of Transcultural Nursing.

[CR18] Kleinman A, Benson P (2006). Anthropology in the clinic: The problem of cultural competency and how to fix it. PLoS Medicine.

[CR19] Knipper M, Seeleman IC, Essink ML (2010). How should ethnic diversity be represented in medical curricula? A plea for systematic training in cultural competence. Tijdschrift voor Medisch Onderwijs.

[CR20] Kumagai AK, Lypson ML (2009). Beyond cultural competence: Critical consciousness, social justice, and multicultural education. Academic Medicine.

[CR21] Kuper A, D’Eon M (2011). Rethinking the basis of medical knowledge. Medical Education.

[CR22] Kutob RM, Bormanis J, Crago M, Harris JM, Senf J, Shisslak CM (2013). Cultural competence education for practicing physicians: Lessons in cultural humility, nonjudgmental behaviors, and health beliefs elicitation. Journal of Continuing Education in the Health Professions.

[CR23] Laan RFJM, Leunissen RRM, Van Herwaarden CLA (2010). The 2009 framework for undergraduate medical education in the Netherlands. Tijdschrift voor Medisch Onderwijs.

[CR24] Leyerzapf H, Abma T, Steenwijk R, Croiset G, Verdonk P (2014). Standing out and moving up: Performance appraisal of cultural minority physicians. Advances in Health Sciences Education. Theory and Practice..

[CR25] Li BU, Caniano DA, Comer RC (1998). A cultural diversity curriculum: Combining didactic, problem-solving, and simulated experiences. Journal of the American Medical Women’s Association.

[CR26] Mackenbach JP, Maas PJ, Bonsel GJ (2008). Volksgezondheid en gezondheidszorg.

[CR27] Mak-van der Vossen M, Peerdeman S, Kleinveld J, Kusurkar R (2013). How we designed and implemented teaching, training, and assessment of professional behaviour at VUmc School of Medical Sciences Amsterdam. Medical Teacher.

[CR28] Maldonado ME, Fried ED, DuBose TD, Nelson C, Breida M (2014). The role that graduate medical education must play in ensuring health equity and eliminating health care disparities. Annals of the American Thoracic Society..

[CR29] McCall L (2005). The complexity of intersectionality. Signs.

[CR30] Muller A (2013). Teaching lesbian, gay, bisexual and transgender health in a South African health sciences faculty: Addressing the gap. BMC Medical Education.

[CR31] Napier AD, Ancarno C, Butler B, Calabrese J, Chater A, Chatterjee H (2014). Culture and health. Lancet.

[CR32] Obedin-Maliver J, Goldsmith ES, Stewart L, White W, Tran E, Brenman S (2011). Lesbian, gay, bisexual, and transgender-related content in undergraduate medical education. JAMA.

[CR33] Patricio M, Harden RM (2010). The Bologna process—A global vision for the future of medical education. Medical Teacher.

[CR34] Pena Dolhun E, Munoz C, Grumbach K (2003). Cross-cultural education in U.S. medical schools: Development of an assessment tool. Academic Medicine.

[CR35] Powell Sears K (2012). Improving cultural competence education: The utility of an intersectional framework. Medical Education.

[CR100] Rademakers JJ, van den Muijsenbergh ME, Slappendel G, Lagro-Janssen AL, Borleffs JC (2008). Sexual harassment during clinical clerkships in Dutch medical schools. Medical Education.

[CR36] Rapp DE (2006). Integrating cultural competency into the undergraduate medical curriculum. Medical Education.

[CR37] Saha S, Guiton G, Wimmers PF, Wilkerson L (2008). Student body racial and ethnic composition and diversity-related outcomes in US medical schools. JAMA.

[CR38] Schiebinger L (2008). Gendered innovations in science and engineering.

[CR39] Shaya FT, Gbarayor CM (2008). The case for cultural competence in health professions education. American Journal of Pharmaceutical Education.

[CR40] Suurmond J, Uiters E, de Bruijne MC, Stronks K, Essink-Bot ML (2011). Negative health care experiences of immigrant patients: A qualitative study. BMC Health Services Research.

[CR41] Tjitra JJ, Leyerzapf H, Abma TA (2011). “Dan blijf ik gewoon stil”: ervaringen van allochtone studenten met interculturalisatie tijdens de opleiding Geneeskunde. Tijdschrift voor Medisch Onderwijs.

[CR42] Tsouroufli M, Rees CE, Monrouxe LV, Sundaram V (2011). Gender, identities and intersectionality in medical education research. Medical Education.

[CR101] Van Wieringen JC, Kijlstra MA, Schulpen TW (2003). [Medical education in the Netherlands: little attention paid to the cultural diversity of patients]. Nederlands tijdschrift voor geneeskunde.

[CR43] Verdonk P (2013). Leerlijn Interculturalisatie en diversiteit.

[CR44] Verdonk P (2015). When I say… reflexivity. Medical Education.

[CR45] Verdonk P, Abma T (2013). Intersectionality and reflexivity in medical education research. Medical Education.

[CR46] Verdonk P, Benschop YW, de Haes HC, Lagro-Janssen TL (2009). From gender bias to gender awareness in medical education. Advances in Health Sciences Education.

[CR47] Vrije Universiteit Amsterdam (2014). VU Onderwijsvisie.

[CR48] Yin RK (2013). Case study research: Design and methods.

[CR49] Young ME, Razack S, Hanson MD, Slade S, Varpio L, Dore KL (2012). Calling for a broader conceptualization of diversity: Surface and deep diversity in four Canadian medical schools. Academic Medicine.

[CR50] Zimmerman MK, Hill SA (2000). Reforming gendered health care: An assessment of change. International Journal of Health Services.

